# Phosphorus-Assisted Biomass Thermal Conversion: Reducing Carbon Loss and Improving Biochar Stability

**DOI:** 10.1371/journal.pone.0115373

**Published:** 2014-12-22

**Authors:** Ling Zhao, Xinde Cao, Wei Zheng, Yue Kan

**Affiliations:** 1 School of Environmental Science and Engineering, Shanghai Jiao Tong University, Shanghai 200240, China; 2 Illinois Sustainable Technology Center, University of Illinois at Urbana-Champaign, Champaign, Illinois 61820, United States of America; Purdue University, United States of America

## Abstract

There is often over 50% carbon loss during the thermal conversion of biomass into biochar, leading to it controversy for the biochar formation as a carbon sequestration strategy. Sometimes the biochar also seems not to be stable enough due to physical, chemical, and biological reactions in soils. In this study, three phosphorus-bearing materials, H_3_PO_4_, phosphate rock tailing (PRT), and triple superphosphate (TSP), were used as additives to wheat straw with a ratio of 1: 0.4–0.8 for biochar production at 500°C, aiming to alleviate carbon loss during pyrolysis and to increase biochar-C stabilization. All these additives remarkably increased the biochar yield from 31.7% (unmodified biochar) to 46.9%–56.9% (modified biochars). Carbon loss during pyrolysis was reduced from 51.7% to 35.5%–47.7%. Thermogravimetric analysis curves showed that the additives had no effect on thermal stability of biochar but did enhance its oxidative stability. Microbial mineralization was obviously reduced in the modified biochar, especially in the TSP-BC, in which the total CO_2_ emission during 60-d incubation was reduced by 67.8%, compared to the unmodified biochar. Enhancement of carbon retention and biochar stability was probably due to the formation of meta-phosphate or C-O-PO_3_, which could either form a physical layer to hinder the contact of C with O_2_ and bacteria, or occupy the active sites of the C band. Our results indicate that pre-treating biomass with phosphors-bearing materials is effective for reducing carbon loss during pyrolysis and for increasing biochar stabilization, which provides a novel method by which biochar can be designed to improve the carbon sequestration capacity.

## Introduction

Turning biomass into biochar through pyrolysis is being actively explored as a tool for long-term carbon sequestration in soil and as a promising strategy to mitigate global warming [1], [2]. The thermal conversion of biomass into biochar is a carbonization process which generally involves an initial carbon loss followed by aromatization. Thus, the carbon sequestration efficiency of biochar depends on both the carbon loss during pyrolysis and the stability of the final product leading to carbon emission over time [3], [4].

In general, over 50% of the carbon present in biomass is lost during pyrolysis due to thermal decomposition and volatilization. This results in low yields of biochar production, especially during the conversion of some plant-based biomasses [5]. Our recent study shows that yields of biochar produced at 500°C from twelve different biomass feedstocks ranged from 27.8% to 58.4%, but the yield of plant-based biomasses such as sawdust or crop wastes was generally low, between 27.8–32.0% [5]. In another study, Hossain et al. (2011) attained biochar yields from sludge ranging from 72.3% to 52.4% with temperatures increasing from 300°C to 700°C [6].

On the other hand, though biochar is regarded as being stable for 1000 years in soil, it still undergoes a slow cumulative degradation, which determines the dispute of biochar as a tool for long-term carbon sequestration. The potential biotic or abiotic mineralization of biochars has been reported by many researchers [6], [7]. Carbon release from abiotic incubations of biochar was 50–90% that of microbially inoculated incubations, and carbon release from both incubations generally decreased with increasing charring temperature [6]. An accelerated aging method which models the long-term stability of biochar indicated that carbon loss ranged between 41.6% and 76.1% [8].

It has been reported that rapid oxidation on the surface of biochar may have important implications for its environmental stability, because aromatic ring structures in biochar may be more available to further microbial decomposition following surface oxidation [9]. Certain chemical substances may be able to strengthen the oxidative resistance of lignocellulosic materials. For example, H_3_PO_4_ is often used during the production of activated carbon because it facilitates the generation of thermally stable phosphorus complexes on the surface of activated carbons (C–O–PO_3_/(CO)_2_PO_2_) [10]. These phosphorus complexes could reduce the reactivity of active sites on carbon and act as a physical barrier for oxygen diffusion in micropores [11], [12]. To the best of our knowledge, no studies have previously been conducted to study this effect on biochar yield and carbon stability of chemical or mineral additives to biomass feedstock prior to pyrolysis.

The overall objective of this study is develop a chemical pre-modification method to improve the potential carbon sequestration capacity of biochar. Thus, three P-bearing substances, including H_3_PO_4_, phosphate rock tailings (PRT), and triple superphosphate (TSP) were used as additives during biochar production. H_3_PO_4_ was chosen because it has been shown to enhance the oxidative resistance of lignin in woodchips [9]. PRT and TSP may coexist with biochar in the soil since these two phosphorus materials are widely used in soil remediation for inactivation of heavy metals [13], [14].

The specific objectives of this study were (i) to determine carbon loss during pyrolysis of the pretreated biomass, (ii) evaluate the effects of these additives on biochar chemical and biological stability, and (iii) to explore the formation mechanisms of these designed biochars.

## Materials and Methods

### Biomass and chemical materials

Wheat straw, a typical plant-based biomass was chosen for this study. Samples were collected from a farm located in Baoshan district in Shanghai, China. No specific field permits were required for this study. The land accessed is not privately owned or protected. No protected species were sampled. All locations used in our study did not involve endangered or protected species. The samples was air-dried to a moisture content of <2% and ground to <1 mm prior to pyrolysis. PRT and TSP are rich in P (14.1% and 20.2%, respectively) and their main constituents are Ca_5_(PO_4_)_3_F and Ca(H_2_PO_4_)_2_·2H_2_O, respectively [15]. PRT is alkaline and slightly soluble in water, while TSP is acidic and highly water soluble [15]. The ground wheat straw was immersed in the water slurry of these materials and mixed homogenously. After 24 h, the pretreated wheat straw was air-dried and put into pyrolysis system to produce biochars [11], [12]. The biomass/additive ratios were within a range of 1: 0.4–0.8: wheat straw/H_3_PO_4_ (g/g) 1: 0.86; wheat straw/PRT (g/g) 1: 0.5; and wheat straw/TSP (g/g) 1: 0.4. The ratios were chosen to ensure that the P composition is enough for mixing with the biomass completely for their full contact in the process of biochar formation.

### Biochar production

Biochar production was conducted in a laboratory-scale pyrolysis system with a stainless steel column of about 3.5 L in a muffle furnace [16]. The production was performed under N_2_ gas with the highest treatment temperature at 500°C. Briefly, about 100 g unmodified or chemically modified wheat straw was weighed into the pyrolyzer, and the heating temperature was then raised at 15°C·min^−1^ to reach four settled gradient temperatures (200°C, 300°C, 400°C and 500°C). At each gradient temperature, the heat temperature was held for 1 h, respectively, to allow enough time for carbonization. Biochar production is an aromatization process, in which volatile compounds are separated from biomass and the rest of carbon is converted to chemically and biological recalcitrant forms. Upon heating, the organic compounds are initially cracked at different temperatures to smaller and unstable fragments. These highly reactive fragments, mainly free radicals with a very short average lifetime, can polymerize into a recalcitrant aromatic structure. Thus it is assumed the gradual temperature enabled biomass components to thoroughly carry out the decomposition and aromatization process. After the pyrolysis treatment was completed, the carbon-rich solid left in the pyrolyzer was the biochar product [5]. For simplicity, the biochars without pre-modification and pre-modified with H_3_PO_4_, PRT, and TSP were referred to as BC, H_3_PO_4_-BC, PRT-BC, and TSP-BC, respectively. The experiments were conducted in three replicates.

### Characterization

Biochar pH was measured in de-ionized water with a solid/liquid ratio of 1∶20 (w/v) after 48 h equilibrium (EUTECH pH510, USA). The elements concentration was measured using an element analyzer (Vario EL III, Elementar, Germany). The bonding environments of P and C atoms (P 2p, C 1s) on the surface of biochar particles were determined using XPS (AXIS Ultra^DLD^, Shimadzu, Japan) with a beam diameter of 200.0 mm and a pass energy of 26 eV. The solid phases of biochar were characterized by X-ray diffraction (D/max-2200/PC, Japan Rigaku Corporation) operated at 35 kV and 20 mA. Data was collected over 2θ range from 10 to 50 using Cu *Kα* radiation with a scan speed of 2^o^ per minute.

### Carbon loss and residue during biochar formation in pyrolysis

Biochar yield (%) was calculated based on the weight of biomass and additives lost during pyrolysis (eq. 1).



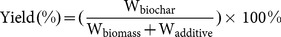
(1)


Carbon loss after pyrolysis was calculated based on yield and total carbon content (eq. 2). 
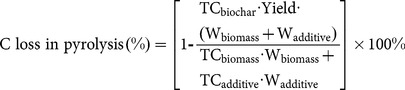
(2)


TC_biomass_, TC_additive_ and TC_biochar_ refer to the total carbon (TC) content in biomass, additive, and biochar, respectively. W_biomass_ and W_additive_ refer to the weight of biomass and additive, respectively; whereas, additives were inorganic substances and contained negligible amounts of TC.

Fixed carbon (FC) represents a relatively stable fraction of carbon in char as calculated by proximate analysis [17]. The FC calculation is shown in eq 3.



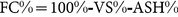
(3)


Volatile solid (VS) is calculated as the weight loss of material under N_2_ atmosphere at 900°C, and ash is evaluated as the weight loss of material under air atmosphere at 900°C.

### Measurement of biochar carbon stability

Three methods were applied to test carbon stability of biochar including heat-resistance stability, oxidation-resistance stability, and microbe-resistance stability. Thermogravimetric analysis (TGA) of the materials under N_2_ and air were used as quick and convenient methods to evaluate the heat-resistance stability and oxidation-resistance stability of each material, respectively [18]. The TGA curves were obtained by heating biochar from 25°C to 900°C at 20°C·min^-1^ on the machine (PerkinElmer Pyris 1 TGA) under both N_2_ flow and atmospheric air. The TGA analysis can simultaneously provide data for analysis of material constituents. For example, the weight loss before 200°C is generally regarded as moisture removal, and the subsequent weight loss can be largely attributed to the decomposition of organic matter [19].

The biological carbon stability of biochar was measured by performing microbial mineralization analysis in sterilized 20 mL borosilicate vials with rubber septa. This method used a simulated microbial soil condition which was widely used in previous reports [7], [9]. Before incubation, the biochars were washed several times to eliminate the volatile smaller molecules from the decomposition of organic matters attached on the surface of the biochars and equalize their initial pH values. For each treatment, three replicate incubations of 0.05–0.30 g of biochar and clean quartz sand with 0.3 mL aqueous nutrient solution [60 g·L^−1^ of (NH_4_)_2_SO_4_ +6 g·L^−1^ of KH_2_PO_4_] were conducted [7]. The addition of sand served to increase permeability, thus increasing the water and oxygen accessibility for the biochar. To culture the bacteria for inoculation, a soil containing bird droppings was taken from a university forest and extracted with deionized water at a 1∶50 solid/liquid ratio (W/V). The supernatant was used for inoculation, and 0.3 mL of inoculation solution was added into the biochar-sand system. The materials were at 30°C. Headspace CO_2_ was measured every 6–10 days using gas chromatography (GC-2010AF, Shimadzu, Japan). Before each measurement, the vials were vacuumed evacuated and 20 mL simulated air (O_2_ and N_2_) with CO_2_ removed was injected into the vials. After 3 days of closed incubation, the released CO_2_ due to decomposition was measured.

## Results and Discussion

### Selected properties of biomass and modified biochars

The compositions of all biochars were presented in Table 1. The C content of H_3_PO_4_-BC and PRT-BC were relative low (28.3% and 29.8%) because of the contribution of the additives residue to the total biochar weight. The N content was a little higher in the PRT-BC and TSP-BC than in other biochars because these two minerals contain a fraction of N. It indicates that the PRT- and TSP-modified biochars may have a better fertility than the unmodified ones. The wheat straw contained a high O content as 41.5%, and biochars contained less O (7.84–29.6%). In H_3_PO_4_-BC, O content is relative high (29.6%), compared with the unmodified biochar (11.6%), which was due to the formation of calcium metaphosphate. P content was 12.5%, 5.98% and 6.78% in the H_3_PO_4_-BC, PRT-BC and TSP-BC, while the value was low in BC. Biochar yields were elevated with the addition of additives, increasing from 31.7% (unmodified biochar) to 40.3%–56.9% (modified biochars) (Table 1). Both PRT-BC and TSP-BC contained higher ash contents (69.3% and 51.9%, respectively) than H_3_PO_4_-BC (25.2%) (Table 1).

**Table 1:** Selected properties of biomass and biochars.

	Wheat straw	BC	H_3_PO_4_-BC	PRT-BC	TSP-BC
pH	6.01	7.77	1.51	8.04	3.89
C (%)	46.8^a^	69.1	28.3	29.8	40.8
H (%)	0.151	0.285	0.023	0.655	0.541
N (%)	0.329	0.691	0.227	1.97	2.03
O (%)	41.5	11.6	29.6	7.84	14.3
P (%)	0.112	0.116	12.5	5.98	6.78
Yield (%)	-	31.7	56.9	54.4	46.9
ASH (%)	4.35	27.1	25.2	69.3	51.9
VS (%)	78.6	13.0	57.6	12.8	12.0
FC (%)	17.1	59.9	17.2	17.9	36.1

PRT: phosphate rock tailing; TSP: triple superphosphate; TC: total carbon; VS: volatile solid; FC: fixed carbon. ^a^ Mean value (n = 3)

The inclusion of different additives in the process of biochar production resulted in changes in their properties (Table 1). The pH value of unmodified biochar was 7.77, falling within the general alkaline pH range of biochars, between 7.5–10.5 [5], [20]. H_3_PO_4_ and TSP addition resulted in biochars with stronger acidity (pH = 1.51 and 3.89, respectively), and PRT-BC was alkaline (pH = 8.04). The pH of the modified biochars likely resulted largely from the acidity or alkalinity of the additive itself.

The physicochemical properties of the modified biochars such as point of zero net charge, cation exchange capacity, specific surface area, etc, were not investigated because carbon residue and carbon stability of biochar are the main focuses of this study. These properties will be investigated in detail in future studies.

### Carbon conversion during pyrolysis

**Figure 1:**
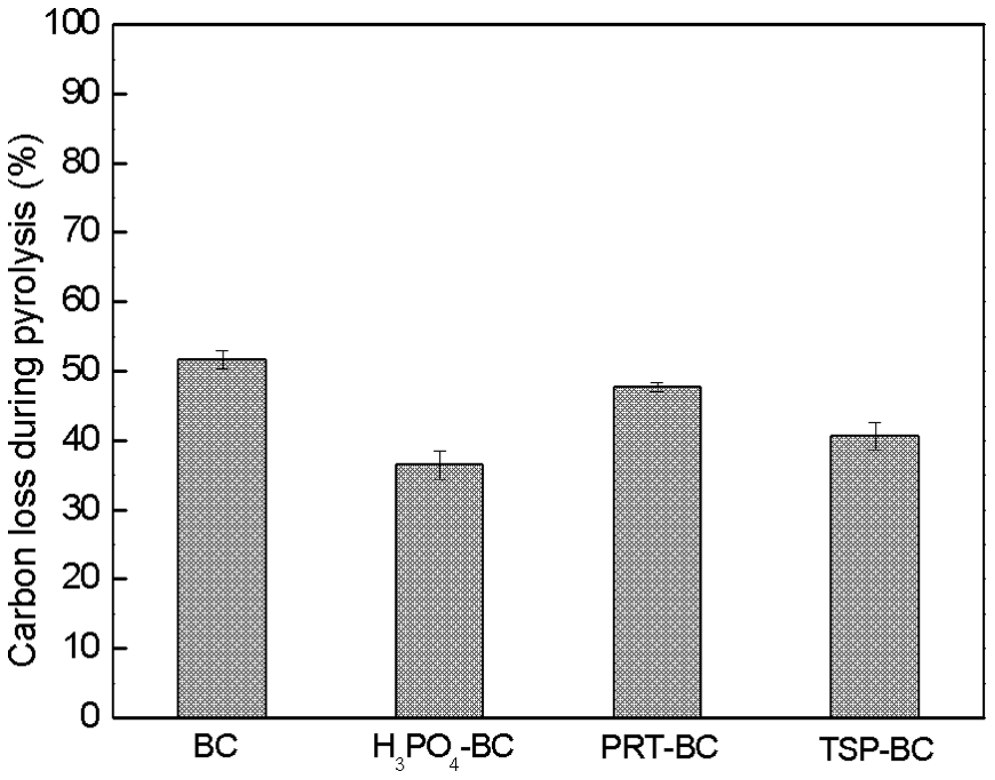
Carbon loss during pyrolysis for biochar production from wheat straw at 500°C with different modifications.

Routine yield which is calculated only from the weight change during the biomass conversion (i.e., eq 1) does not represent the real carbon-negativity of biomasses; thus, the entire carbon budget was evaluated in this study. The total carbon loss calculated from eq. 2 is presented in Fig. 1. There was 51.7% carbon loss during wheat straw conversion into biochar. However, addition of all P-containing chemicals greatly alleviated the carbon loss to 35.5–47.7%, with the carbon loss reduced by 7.74–31.4% compared to the unmodified biochar. The underlying mechanisms for carbon loss alleviation will be discussed in the next sections. The extent of the alleviation may differ with different feedstock and production conditions, which needs separate investigation.

The FC content of the biochars is presented in Table 1. FC is a fraction of biochar and represents a relatively stable fraction in biochar which enables it to be the “sequestrated C”. FC partially originates from feedstock carbon, but it is also a conversion product from pyrolysis processes [20]. FC was calculated as the non-ash fraction that could be burned in air but not be lost in N_2_ at 900°C. The FC values for H_3_PO_4_-BC, PRT-BC and TSP-BC were 17.2%, 17.9% and 36.1%, respectively, which were lower than that of the control BC (59.9%). Note that the relative low FC values were due to the high ASH content in PRT-BC and the high VS fraction in H_3_PO_4_-BC.

### Thermal and oxidation stability of modified biochars

**Figure 2:**
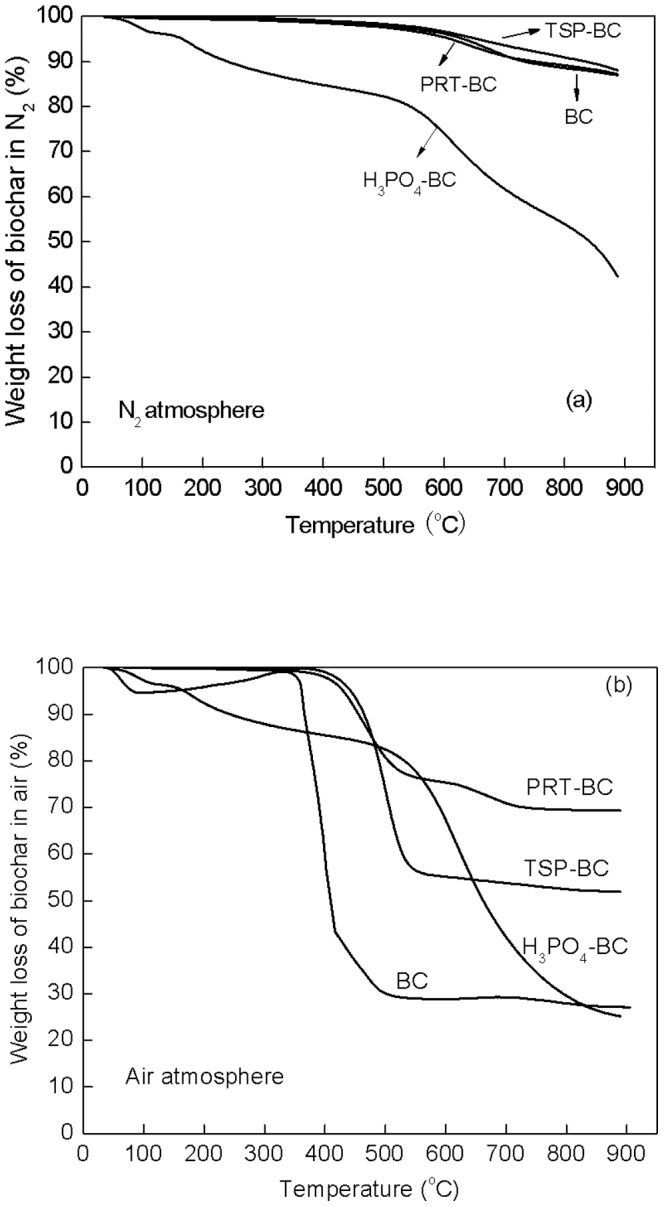
TGA curves of the biochars in N_2_ (a) and air (b) atmosphere.

TGA curves of biochars over the temperature ranging from 25°C to 900°C under N_2_ conditions are shown in Fig. 2a, which reflects the thermal stability of biochar. The modified biochars had similar TGA curves to the unmodified biochar. The majority of weight loss occurred from 600°C to 900°C after a minor weight loss from 200°C to 500°C (Fig. 2a). H_3_PO_4_-BC showed a larger initial weight loss than the other biochars. The main weight loss of biochar was most likely due to the decomposition of either organic fractions of the carbon skeleton or some combination of carbon and additives (see section 3.5). Overall, there was not much difference in the temperature at which the main weight loss occurred between the unmodified and modified biochars, indicating that these additives had no influence on the thermal stability of biochars.

The mass loss of biochars in air is generally related to their oxidative stability, and it is believed that the more stable a substance is, the higher the temperature needs to be for decomposition [21]. Fig. 2b shows TGA curves of biochars under normal air conditions. All the modified biochars decomposed at higher temperatures (490°C–650°C) than the unmodified ones (400°C), especially the H_3_PO_4_-BC (650°C). The results indicate that these additives could increase the oxidative stability of biochar. Significant oxidation inhibition by phosphorus has also been observed in many other carbon materials production [22], [23], [24] and possible mechanisms will be discussed in section 3.5.

### Microbe-resistance stability of modified biochars

**Figure 3:**
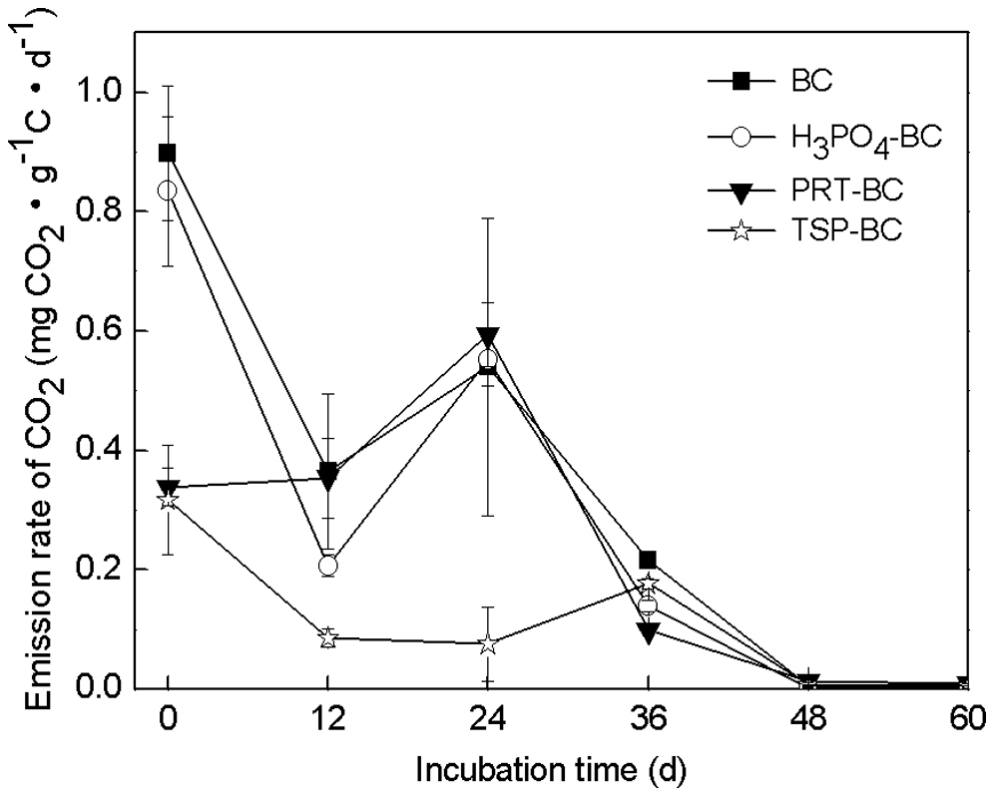
Emission rates of CO_2_ from the biochars during aerobic incubation.

The emission rate of CO_2_ from biochar during aerobic incubation represents its biological mineralization stability [25], [26]. The pH of these materials fluctuated in the range of 5.5–6.5, which was appropriate for the microbial activity. All additives were observed to reduce the CO_2_ emission rate from biochar to some extent (Fig. 3). TSP-BC showed the lowest mineralization rate. The decrease in mineralization rate by H_3_PO_4_ and PRT was also significant. The majority of change in the CO_2_ emission rates occurred within the first 36 days. After the 48^th^ day, the CO_2_ emission of all samples decreased to a very low level, and the differences among the unmodified and modified biochar seemed unremarkable. However, because biochar can be oxidized in the soil for many years, small differences in CO_2_ emissions may have a significant effect if summed over a long period of time, thus, long-term stability of modified biochar needs further investigation. Note that the evaluation of biological stability was made in this work using a simulated microbial soil condition [7], [9]. However, the stability of biochar may also be influenced by soil properties such as soil pH, soil carbon content and the presence of other organic substances. Therefore, incubation tests of modified biochar in a real soil system should be conducted in a future study [27].

The cumulative CO_2_ emission amount during the 60-day aerobic incubation was calculated according to the average emission rates every 12 days, which were 18.9, 15.8, 14.8 and 6.09 mg CO_2_· g C^−1^ for BC, H_3_PO_4_-BC, PRT-BC, and TSP-BC, respectively. H_3_PO_4_, PRT, and TSP treatments reduced microbial CO_2_ emission of biochar by 16.4%, 21.7%, and 67.8%, compared to unmodified biochar. Many studies concluded that the main CO_2_ emission occurred in the first 60 days [7], [28]; thus, the obvious decrease in CO_2_ emission during the 60-day incubation period in this study suggests that the three additives could enhance the biological mineralization stability of biochar. The effectiveness followed the trend TSP>PRT>H_3_PO_4_.

### Interference of phosphorus on biomass pyrolysis

**Figure 4:**
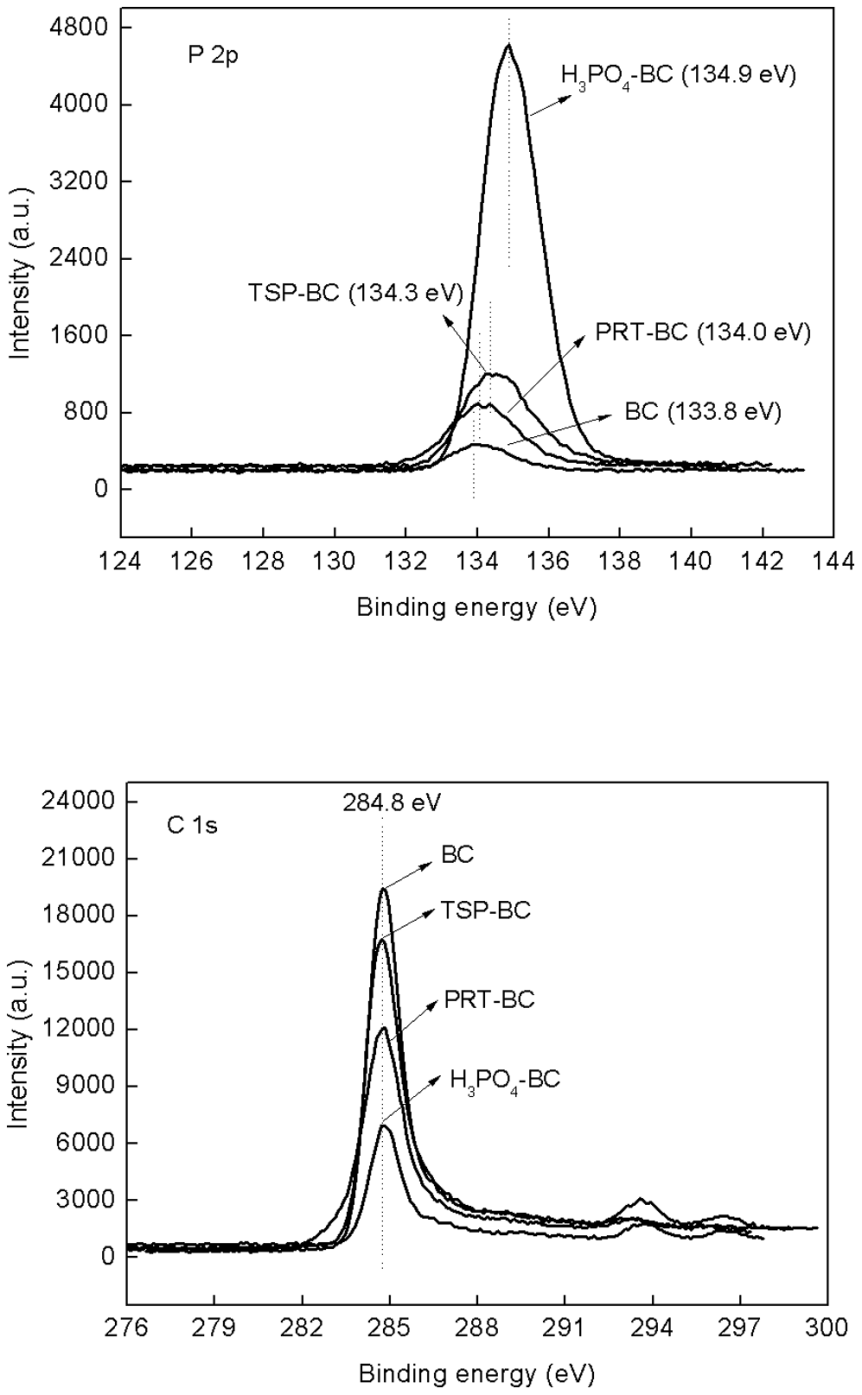
XPS spectra of P 2p and C 1s electron for the unmodified and modified biochars.

It is not surprising that inorganic substances have influence on the charcoal yield, product distribution, or product selectivity during pyrolysis [29]–[32]. For example, iron improved the O-containing functional groups of biochar [33]. In this study, the exact reactions between the additives and wheat straw cannot yet be determined, but potential mechanisms of modification effects on biochar carbon residue and stability could be proposed according to the results obtained from this study. Fig. 4 shows the XPS spectra of P 2p and C 1s electron (spin-orbit) for the unmodified and modified biochars. The additives made the peak of P 2p shift from low binding energy (BC: 133.8 eV) to higher binding energy (H_3_PO_4_: 134.9 eV; PRT: 134.0 eV; TSP: 134.3 eV). The binding energy intensity of the main C 1s peak at 284.8 eV was almost not changed (Fig. 4b), while the height of the peaks was decreased by the introduction of the additive materials. This indicates that the surface C content was reduced.

**Figure 5:**
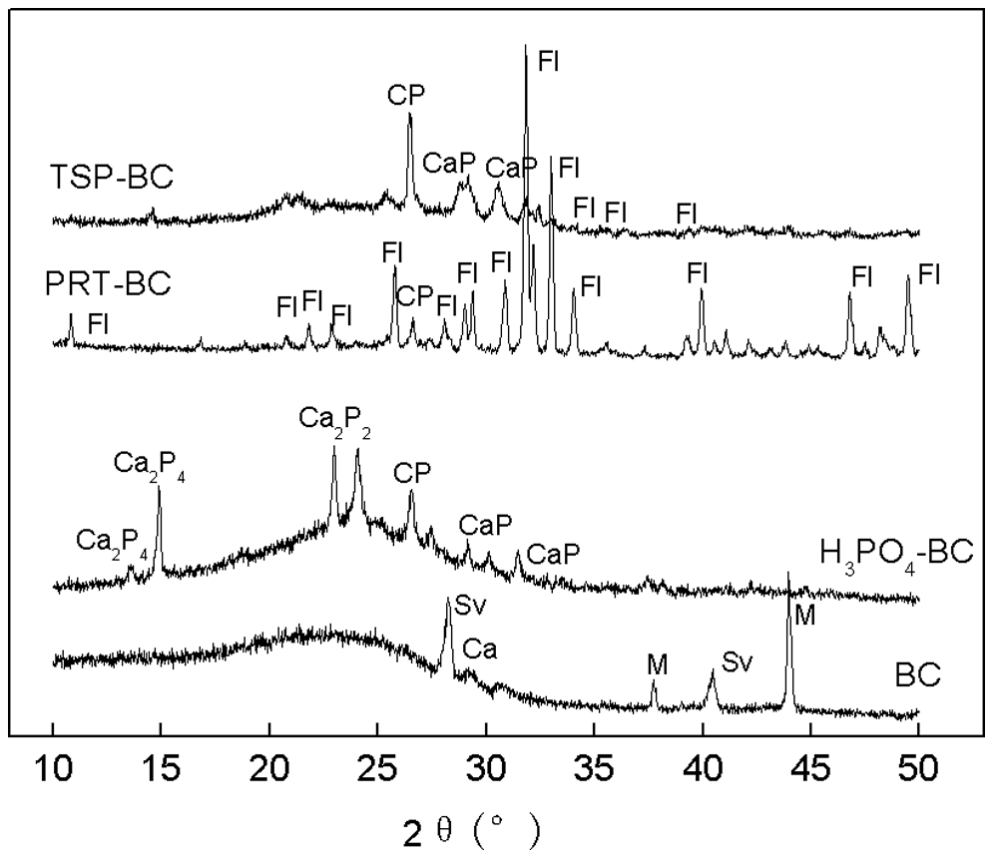
X-ray diffraction (XRD) patterns of the unmodified and modified biochars.

A P 2p peak around 135.0 eV is generally assigned to metaphosphate or C-O-PO_3_ type groups [34], [35]. Addition of chemicals, especially H_3_PO_4_, increased the P 2p peak from 133.8 eV in unmodified BC to about 135 eV, indicative of the P-C compounds formation. This observation agrees with previous findings that H_3_PO_4_ addition mainly resulted in the formation of oxygen-containing phosphorus groups which may include metaphosphates, C–O–PO_3_ groups, or C–PO_3_ groups [34], [35]. These groups are suggested to act as a physical barrier against carbon decomposition, as well as to block the active carbon sites [22], [18], resulting in reduced oxidation and mineralization of biochar. Formation of P-C compounds was further evidenced by X-ray diffraction (XRD) analysis (Fig. 5). Compared to the unmodified BC, a new peak at 2θ^0^ = 26.6, most likely corresponding to the P-C compounds was observed in the H_3_PO_4_-BC and TSP-BC. Although PRT is also rich in P, it is less soluble, allowing it behave differently from soluble TSP and H_3_PO_4_. A weak peak of P-C compounds was observed at 2θ^0^ = 26.6 in the PRT-BC (Fig. 5). Qian et al. (2014) pointed out that the P-containing radicals react with the aromatic rings produced by the pyrolysis of lignin to form P-containing species, which is an important factor influencing the distribution and stabilization of P in char [36]. Uchimiya and Hiradate (2014) indicated that orthophosphate such as CH_3_−O−PO_3_^2−^ and phenyl−O−PO_3_^2−^ formed in pyrolysis were stable [37]. Klupfel et al. (2014) also proposed that biochar has redox properties and acts as electron-donating [38]. This indicated that P has potential to react with the carbon in biochar. Overall, all three P-bearing additives induced the formation of P-C compounds.

According to the results of this study, the P-bearing additives seemed to have no influence on the thermal stability of biochar products, but they did improve oxidative stability and biological degradation. Thermal stability is a function of bond energy, while both the incineration of volatile matters and the carbon to CO_2_ conversion require contact between C and O_2_ or bacteria. Thus it could be speculated that the bond energy of the reaction products from additives is low, and these additives could also form a physical layer to hinder the contact of C with O_2_ or bacteria or occupy the active sites of the carbon band.

### Implications in application of biochar as a carbon fixer

Although biochar has been considered as a multi-functional material for improving the environment in many different ways, its primary environmental significance has always been carbon sequestration [39], [40]. However, challenges for the effectiveness of biochar as a carbon fixer still remain. About half of the initial carbon from the biomass is not converted to biochar during pyrolysis, and the final biochar product is still not as stable as necessary for long-term carbon sequestration, which causes biochar to remain a controversial carbon fixer [41].

This study presents a novel idea that biochars with high carbon content residue and stability can be designed using pre-modification during the feedstock preparation. The P-containing materials chosen did show a tendency to act as passivators to reduce carbon loss during pyrolysis and enhance the stability of biochar, though the two effect extents were not consistent with one specific additive. The potential capacity of carbon sequestration is expected to be improved through the regulation of process conditions. Additionally, these modifications might change biochar's physicochemical properties, which may affect the soil environment when biochar is applied into soil as a carbon sequestration tool. Additional benefits to the soil may also be obtained; for example, the modified PRT-BC and TSP-BC contain high P which may be effective in immobilizing heavy metals in contaminated soils [42], [43]. Of course, it must be noted that the low pH value of the H_3_PO_4_-BC biochar may limit its soil application in acidic soils.

Overall, there is some promise that biochar can be designed to provide multi-win effects, i.e., carbon sequestration, soil improvement, and contamination remediation.

## Conclusions

During the charring process of biomass to biochar, over 50% carbon is typically lost, and the generated biochar may not be stable enough in soil due to physical, chemical, and biological reactions. In this study, the biomass precursor was pre-treated using three P-bearing chemical substances including H_3_PO_4_, PRT and TSP, aiming to reduce carbon loss during charring and simultaneously increase the carbon stability of the final biochar product. The results show that these chemicals reduced the carbon loss, improved the oxidation stability and reduced the microbial mineralization. This study put forward the novel idea that people can design biochar to improve its carbon residue and stability through passivator addition during feedstock preparation, which enables it to be a prospective tool for carbon sequestration.
